# Hsa-miR-3178/RhoB/PI3K/Akt, a novel signaling pathway regulates ABC transporters to reverse gemcitabine resistance in pancreatic cancer

**DOI:** 10.1186/s12943-022-01587-9

**Published:** 2022-05-10

**Authors:** Jianyou Gu, Wenjie Huang, Xianxing Wang, Junfeng Zhang, Tian Tao, Yao Zheng, Songsong Liu, Jiali Yang, Zhe-Sheng Chen, Chao-Yun Cai, Jinsui Li, Huaizhi Wang, Yingfang Fan

**Affiliations:** 1 Department of Hepatobiliary Surgery I, Zhujiang Hospital, Southern Medical University, Guangzhou, Guangdong People’s Republic of China; 2 Institute of Hepatopancreatobiliary Surgery, Chongqing General Hospital, Chongqing, People’s Republic of China; 3 Department of Pharmaceutical Sciences, College of Pharmacy and Health Sciences, St. John’s University, Queens, NY 11439 USA

**Keywords:** Hsa-miR-3178, RhoB, Gemcitabine resistance, PI3K/Akt, Pancreatic cancer, ABC transporters

## Abstract

**Background:**

Although gemcitabine has been considered as the first-line drug for advanced pancreatic cancer (PC), development of resistance to gemcitabine severely limits the effectiveness of this chemotherapy, and the underlying mechanism of gemcitabine resistance remains unclear. Various factors, such as ATP binding cassette (ABC) transporters, microRNAs and their downstream signaling pathways are included in chemoresistance to gemcitabine. This study investigated the potential mechanisms of microRNAs and ABC transporters related signaling pathways for PC resistance to gemcitabine both in vivo and in vitro.

**Methods:**

Immunohistochemistry and Western blotting were applied to detect the expression of ABC transporters. Molecular docking analysis was performed to explore whether gemcitabine interacted with ABC transporters. Gain-of-function and loss-of-function analyses were performed to investigate the functions of hsa-miR-3178 in vitro and in vivo. Bioinformatics analysis, Western blotting and dual-luciferase reporter assay were used to confirm the downstream regulatory mechanisms of hsa-miR-3178.

**Results:**

We found that P-gp, BCRP and MRP1 were highly expressed in gemcitabine-resistant PC tissues and cells. Molecular docking analysis revealed that gemcitabine can bind to the ABC transporters. Hsa-miR-3178 was upregulated in gemcitabine resistance PANC-1 cells as compared to its parental PANC-1 cells. Moreover, we found that hsa-miR-3178 promoted gemcitabine resistance in PC cells. These results were also verified by animal experiments. RhoB was down-regulated in gemcitabine-resistant PC cells and it was a downstream target of hsa-miR-3178. Kaplan–Meier survival curve showed that lower RhoB expression was significantly associated with poor overall survival in PC patients. Rescue assays demonstrated that RhoB could reverse hsa-miR-3178-mediated gemcitabine resistance. Interestingly, hsa-miR-3178 promoted gemcitabine resistance in PC by activating the PI3K/Akt pathway-mediated upregulation of ABC transporters.

**Conclusions:**

Our results indicate that hsa-miR-3178 promotes gemcitabine resistance via RhoB/PI3K/Akt signaling pathway-mediated upregulation of ABC transporters. These findings suggest that hsa-miR-3178 could be a novel therapeutic target for overcoming gemcitabine resistance in PC.

**Supplementary Information:**

The online version contains supplementary material available at 10.1186/s12943-022-01587-9.

## Introduction

Pancreatic cancer (PC) is a malignant tumor with a very poor prognosis. Its five-year survival rate is only around 10% [1]. Gemcitabine is the cornerstone drug of PC treatment in all stages of this disease [2]. However, therapeutic effect of gemcitabine is hampered by drug resistance [3]. The mechanisms of gemcitabine resistance in pancreatic cancer are complex. Whether gemcitabine resistance was associated with the ATP-binding cassette (ABC) transporters is not fully studied [4]. ABC transporters are a superfamily consisting of seven (A to G) subfamilies with a total of 48 members, and the overexpression of these transporters is the main mechanism leading to multidrug resistance (MDR) of tumor chemotherapy [5]. However, little is known about their role in gemcitabine resistance.

Recent studies on the involvement of microRNAs (miRNAs) in chemoresistance may offer new opportunity to overcome the chemotherapeutic resistance of gemcitabine in PC [6–8]. MiRNAs are a category of small endogenous single-stranded RNAs usually play a negative regulatory role by targeting specific mRNAs for degradation or translation suppression [9, 10]. Aberrant expression of miRNAs is linked to pathological processes in various cancers, such as cell proliferation, apoptosis, invasion, metabolism, differentiation and drug resistance [11–15]. In our previous study, four differentially expressed miRNAs, including hsa-miR-3178, hsa-miR-485-3p, hsa-miR-574-5p, and hsa-miR584-5p, were identified in gemcitabine-resistant PC cells versus parental cells. Among them, hsa-miR-3178 significantly affected the survival of pancreatic cancer patients (HR = 2.27, Logrank *P* = 0.0018) [16], but its downstream signaling pathway and the underlying mechanism remain unclear.

Ras Homolog Family Member B (RhoB) belongs to the small GTPases family. The Rho subfamily of RhoGTPases plays critical roles in physiological and pathological processes of cancers, and consists of three proteins, RhoA, RhoB, and RhoC [17]. RhoB is localized at endosomes as well as at the plasma membrane, endosomes, Golgiassociated vesicles and the nucleus[18, 19]. RhoB is an immediate early response gene that is induced by a variety of stimuli [18]. RhoB has been reported to be targeted by various miRNAs to regulate apoptosis, proliferation and migration of cancer cells [20, 21]. Moreover, as a tumor suppressor, downregulation of RhoB is associated with drug resistance in cancers [22, 23]. In pancreatic cancer, the relationship between RhoB and gemcitabine resistance remain elusive.

In this study, we found that RhoB is one of the downstream target molecules of hsa-miR-3178. Hsa-miR-3178/RhoB axis upregulates the expression of ABC transporters via activating PI3K/Akt signaling pathway to induce gemcitabine resistance in PC.

## Materials and methods

### Cell cultures and reagents

Pancreatic cancer cell lines, AsPC-1, BxPC-3, CFPAC-1, MIA PaCa2, SW1990 (Shanghai Institute of Biochemistry and Cell Biology, China), and Hs766t (ATCC, Manassas, VA, USA), and an immortalized human pancreatic ductal epithelial cell line: HPDE6-C7 (BeNa Culture Collection, China), were used in this study. The acquisition and culture of PC gemcitabine resistant cells (PANC-1-GEM) and their parental sensitive cells (PANC-1) was the same as described in our previous article [16]. The PI3K inhibitor LY294002 (10 μM, MCE, USA) and PI3K agonist 740Y‐P (30 μM, MCE, USA) were used to treat cells for 24 h. Gemcitabine were purchased from Selleck Chemicals Co. Ltd (Houston, TX, USA).

### Tissue Specimens and Immunohistochemistry (IHC)

The expression of ABC transporter proteins was analyzed by immunohistochemical staining (IHC). A tissue microarray containing 87 cases of pathologically diagnosed PC and 3 gemcitabine-resistant PC tissues and 3 gemcitabine-sensitive tissues were used. Another tissue microarray containing 70 PC and 58 adjacent nontumor tissues were used. Additionally, 15 pairs of primary fresh-frozen PC tissue samples and matched adjacent non-tumor tissues, 13 fresh PC tissues and non-tumor tissues were taken for qRT-PCR analysis. The tissues were obtained from the Institute of Hepatopancreatobiliary Surgery, Southwest Hospital, Army Medical University in Chongqing, China. The ethics committee of Southwest Hospital approved the use of the clinical specimens.

IHC was performed according to the manufacturer’s instructions (Maixin, Fuzhou, China). The IHC score was calculated on the basis of staining intensity and percentage of positively stained cells. The staining intensity was scored as follows [24]: 0 (no staining), 1 (weak staining), 2 (moderate staining), and 3 (strong staining). The percentage of positively stained was scored as follows: 0 (no staining), 1 (1–10%), 2 (10–50%), 3 (more than 50%). The staining index (SI) was calculated by multiplying the values of staining intensity and percentage of positively stained cells. Using this method of assessment, tumor samples were divided into groups with low (⩽ 4) or high expression (⩾ 6) of the target proteins.

### Western blotting

Western blotting was performed as previously described [25] using antibodies against RhoB (1:500; Proteintech, USA), phospho-Akt 308, phospho-Akt 473 and Akt (1:1,000; Cell Signaling Technology, USA), phospho-PI3K (1:1,000; Abcam, USA), PI3K (1:1,000; Cell Signaling Technology, USA), P-glycoprotein (P-gp, also known as ABCB1) (1:500; Proteintech, USA), breast cancer resistance protein (BCRP, also known as ABCG2) (1:500; Proteintech, USA), multidrug resistance-associated protein 1 (MRP1, also known as ABCC1) (1:500; Abcam, USA) and β-actin (1:5,000; Cell Signaling Technology, USA). The secondary antibody used is horseradish peroxidase-conjugated antibody (anti-rabbit; 1:5,000; Cell Signaling Technology). Protein levels were normalized to the endogenous control β-actin.

### In silico* molecular docking analysis*

The structure of gemcitabine was optimized for docking analysis using the Maestro V11.1 (Schrodinger, LLC, New York, 2020). The hydrogenation and force field structure were optimized by removing water molecules from the P-gp (PDB ID: 6FN1) [26], BCRP (PDB ID: 6FFC) [27] and MRP1 (PDB ID: 5UJA) [28] receptor model structures. The Glide module of Maestro software was used to dock the protein receptor and ligand in an induced-fit method. The cavity of 20 Å was selected as the docking active region, and the docking calculation was carried out with standard parameters. The method of induced-fit can make the gemcitabine adopt an optimal conformation to bind to the protein receptor, and the protein receptor will also change the original conformation to better bind to the gemcitabine [29]. The docking models with the highest score were analyzed and visualized.

### RNA extraction and real-time PCR

Methods and reagents for RNA extraction and qRT-PCR experiments were the same as described in our previous article [16]. The bulge-loop miRNA qRT-PCR primer sets specific for hsa-miR-3178 were designed and synthesized by RiboBio Inc (Guangzhou, China). The RhoB primer with sequences 5'-GCGGTAGGCGTGTACGGT-3' (forward) and 5'-CTGGAATAGCTCAGAGGC-3' (reverse) were synthesized by the GeneCopoeia Inc (Guangzhou, China).

### Overexpression and silencing of genes

Agomir-3178 (hsa-miR-3178 mimics, 30 nM), mimic negative control (MNC), antagomir-3178 (hsa-miR-3178 inhibitor, 30 nM), inhibitor negative control (INC), RhoB siRNA (50 nM), scramble control (Scr) and overexpression Lentiviral (pReceiver-Lv216-CMV-1Flag-SV40-Puromycin) were obtained from GeneCopoeia Inc (Guangzhou, China). The transfection reagent used was Lipofectamine 3000 (Invitrogen, USA).

### Luciferase reporter assay

The wild-type and mutant 3’ UTR sequence of RhoB were inserted into the SI-Check2 vector at a position downstream of the SV40 promoter. Mutations were generated in the binding sites of the 3’ UTR. Next, 0.16 µg plasmid containing the RhoB-3’UTR/RhoB-3’UTR mutant and 5 pmol hsa-miR-3178/Negative Control (NC) was transfected into 293 T cells, PANC-1 cells and PANC-1-GEM cells. Firefly luciferase (internal reference) and Renilla luciferase activities at 48 h after transfection were detected using the Promega Dual-363 Luciferase system according to the manufacturer’s instructions.

### Cell viability assay

Cell viability was determined by Cell Counting Kit 8 (CCK-8) assay. The specific steps and reagents used were the same as described in our previous article [16]. Cells were harvested and resuspended, and seeded in a 96-well plate at 5 × 10^3^ cells per well. After incubating for 24 h, gemcitabine was added. After 72 h of incubation, CCK-8 (10 µl) was added to each well and the cells were further incubated for 2 h before detection. The half-inhibitory concentration (IC_50_) of anticancer drug, at which 50% of cells were inhibited, was calculated as previously described [30].

### Cell proliferation analysis

The viability and proliferation of PC cells was determined using the CCK-8 assay and the 5-Ethynyl-2’-deoxyuridine (EdU) immunofluorescence assay (RiboBio, Guangzhou, China) after cells were treated with gemcitabine (1 μmol/L, PANC-1 cells; 150 μmol/L, PANC-1 cells) for 72 h. For CCK-8 assay, 5 × 10^3^ cells with or without transfection were seeded in 96-well plates and 10 µl of Cell Counting Kit-8 solution were added to each well and incubated for 2 h at 37 °C before detection. The OD value was detected at 24, 48, 72, and 96 h after the cells were seeded. For EdU assay, 5 × 10^3^ cells with or without transfection were seeded in 96-well plates. After incubation for 72 h (gemcitabine, 1 μmol/L, PANC-1 cells; 150 μmol/L, PANC-1 cells), the cells were incubated with EdU and immunofluorescence staining was performed.

### Apoptosis assays

Cellular apoptosis was quantified by flow cytometry using an Annexin V-FITC/PI Staining kit according to the manufacturer’s instruction (KeyGEN BioTECH, Jiangsu, China). Briefly, after treatment with gemcitabine (1 μmol/L, PANC-1 cells; 150 μmol/L, PANC-1 cells) for 72 h, 5 × 10^5^ cells were harvested and suspended in 500 μl of binding buffer containing 5 μl annexin V-FITC and 5 μl PI. Then cells were incubated for 15 min at room temperature in the dark. The apoptotic cells were detected by a FACS Calibur (BD Biosciences).

### Animal experiments

Four to six weeks female BALB/c nude mice were purchased from the Peking University Animal Center (Beijing, China). The mice were randomized into 6 subgroups (*n* = 4/group). The mice were inoculated subcutaneously in the left oxter with 5 × 10^6^ PANC-1 cells (transfected with agomir-3178, agomir negative control or PBS) or PANC-1-GEM cells (transfected with antagomir-3178, antagomir negative control or PBS). One week after injection, when the mice had a palpable tumor with a diameter of ~ 2 mm, gemcitabine (5 mg/kg) was administered intraperitoneally every three days. Tumor length (L) and width (W) were monitored every week using a digital Vernier caliper and tumor volume was determined using the equation (L*W^2^)/2. The mice were sacrificed 4 weeks post injection and tumors were surgically removed, weighed and analyzed by hematoxylin–eosin staining (HE) and IHC. A TUNEL assay was performed on paraffin-embedded tissue sections according to the manufacturer’s instructions (Servicebio). The animal study was approved by the Ethics Committee of Southwest Hospital.

### Statistical analysis

Statistical analyses were performed using SPSS statistical software (version 22.0, Chicago, IL, USA). Data were presented as the mean ± standard deviation of three independent experiments. Statistical tests for data analysis included Chi-square test, log-rank test, Student’s t-test, and ANOVA with Turkey’s method. All tests were two-sided and statistical significance was defined as *p* < 0.05. Figures were generated using GraphPad Prism 8 Software.

## Results

### P-gp, BCRP and MRP1 proteins were overexpressed in gemcitabine-resistant pancreatic cancer tissues and cells.

**Fig. 1:**
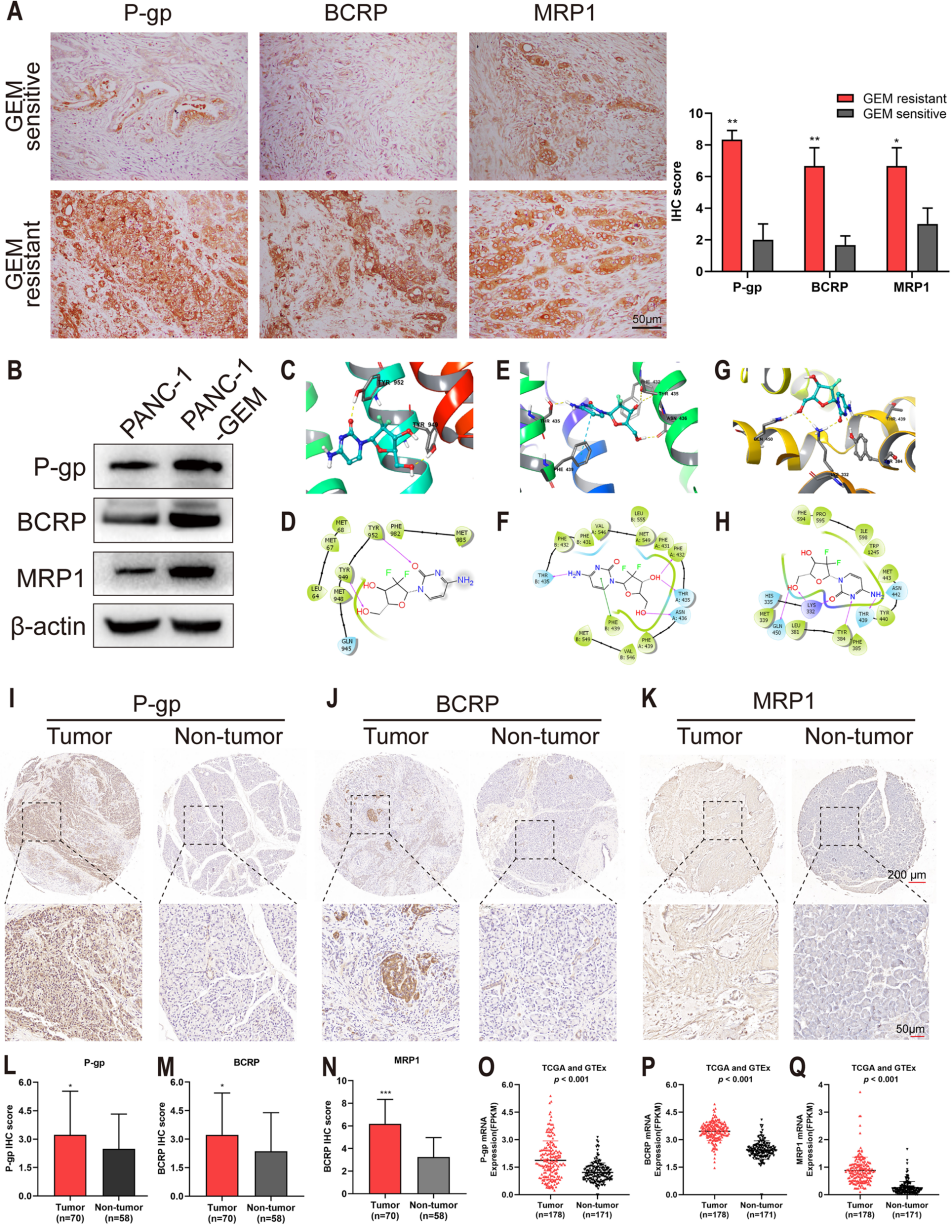
P-gp, BCRP and MRP1 were overexpressed in gemcitabine-resistant pancreatic cancer tissues and cells. Docking simulation revealed gemcitabine binds to the substrate binding site of P-gp, BCRP and MRP1 proteins. **A** IHC staining of P-gp, BCRP, MRP1 in pancreatic cancer gemcitabine-resistant and gemcitabine-sensitive tissues, 200x. Scale bar, 50 μm. **B** Western blotting on the expression of P-gp, BCRP, MRP1 in the indicated cells. β-actin was used as a loading control. **C-H** Interaction between gemcitabine and human P-gp, BCRP and MRP1 protein models. Details of the best-scoring pose of gemcitabine in the drug binding pocket of P-gp (**C**), BCRP (**E**) and MRP1 (**G**) binding pocket. 2D diagram of the interaction between gemcitabine and P-gp (**D**), BCRP (**F**) and MRP1 (**H**) binding pocket. The cavity of 20 Å was selected as the docking active region, and the docking calculation was carried out with standard parameters. Purple arrows represent hydrogen bonding, and green dash lines represent pi-pi interactions. **I-N** IHC staining demonstrated the P-gp (**I, L**), BCRP (**J, M**) and MRP1 (**K, N**) overexpression in PC tissues compared to the pancreatic non-tumor tissues. **O-Q** The mRNAs expression profile showed P-gp (**O**), BCRP (**P**) and MRP1 (**Q**) overexpression in pancreatic cancer tissue as compared to non-tumor tissue obtained from the public database (TCGA and GTEx). Data are presented as mean ± SD; *P < 0.05. IHC: immunohistochemistry

ABC transporter superfamily has seven (A to G) subfamilies with a total of 48 members, and is a special class of proteins in the cell membrane [31]. Studies have shown that ABC transporters play an important role in gemcitabine chemotherapy resistance in PC [31, 32]. Among them, P-gp, BCRP and MRP1 are the recognized molecules that contributed to the development of multidrug resistance [4, 33]. In this study, IHC staining further confirmed that P-gp, BCRP, MRP1 were overexpressed in gemcitabine-resistant compare with gemcitabine-sensitive tissues (Fig. 1 A). Western blotting also showed that their expressions in gemcitabine-resistant PANC-1-GEM cells were higher than that in gemcitabine-sensitive PANC-1 cells (Fig. 1 B). The expression levels of P-gp, BCRP, and MRP1 were also markedly upregulated in pancreatic cancer tissues as compared to the pancreatic non-tumor tissues (Figs. 1I-N). These findings were validated by data from the Cancer Genome Atlas dataset (TCGA) and the Genotype-Tissue Expression (GTEx) (Fig. 1O-Q).

### Gemcitabine binds to the substrate binding site of P-gp, BCRP and MRP1 proteins

Molecular docking simulation was performed to explore whether gemcitabine interacted with P-gp, BCRP and MRP1 proteins. The docking analysis showed that gemcitabine binds to these ABC transporter proteins. The affinity scores of interactions between gemcitabine and P-gp, BCRP and MRP1 are -8.568, -9.402 and -10.116 kcal/mol, respectively, indicating that gemcitabine has strong binding affinity with P-gp, BCRP and MRP1. It was found that the hydroxyl group of gemcitabine acts as the hydrogen bond receptor with the amino acid residue Tyr949, while the carbonyl group acts as the hydrogen bond receptor with the amino acid residue Tyr952 and forms the hydrogen bond interaction with P-gp. Moreover, the amino acid residues Leu64, Met67, Met68, Met948, Phe982 and Met985 on the binding domain of P-gp were hydrophobic with gemcitabine, which enhanced the interaction between gemcitabine and P-gp. Gemcitabine formed hydrogen bonds with amino acid residues Phe432, Thr435 and Asn436 of BCRP, and formed π-π interaction with Phe439. Amino acid residues Phe431, Phe439, Val546 and Met549 on the BCRP binding domain were hydrophobic with gemcitabine, which enhanced the interaction between gemcitabine and BCRP. Gemcitabine forms hydrogen bonds with amino acid residues Lys332, Tyr384, Thr439 and Gln450 of MRP1 receptor. Also, the amino acid residues Met339, Leu381, Phe385, Tyr440, Met443, Phe594, Pro595, Ile598 and Trp1245 on the MRP1 binding domain were hydrophobic with gemcitabine, which enhanced the interaction between gemcitabine and MRP1 (Fig. 1 C-H). These results suggested that gemcitabine possessed high binding affinity with P-gp, BCRP and MRP1.

### Overexpression of hsa-miR-3178 promoted gemcitabine resistance in PANC-1 cells both in vitro and in vivo

**Fig. 2:**
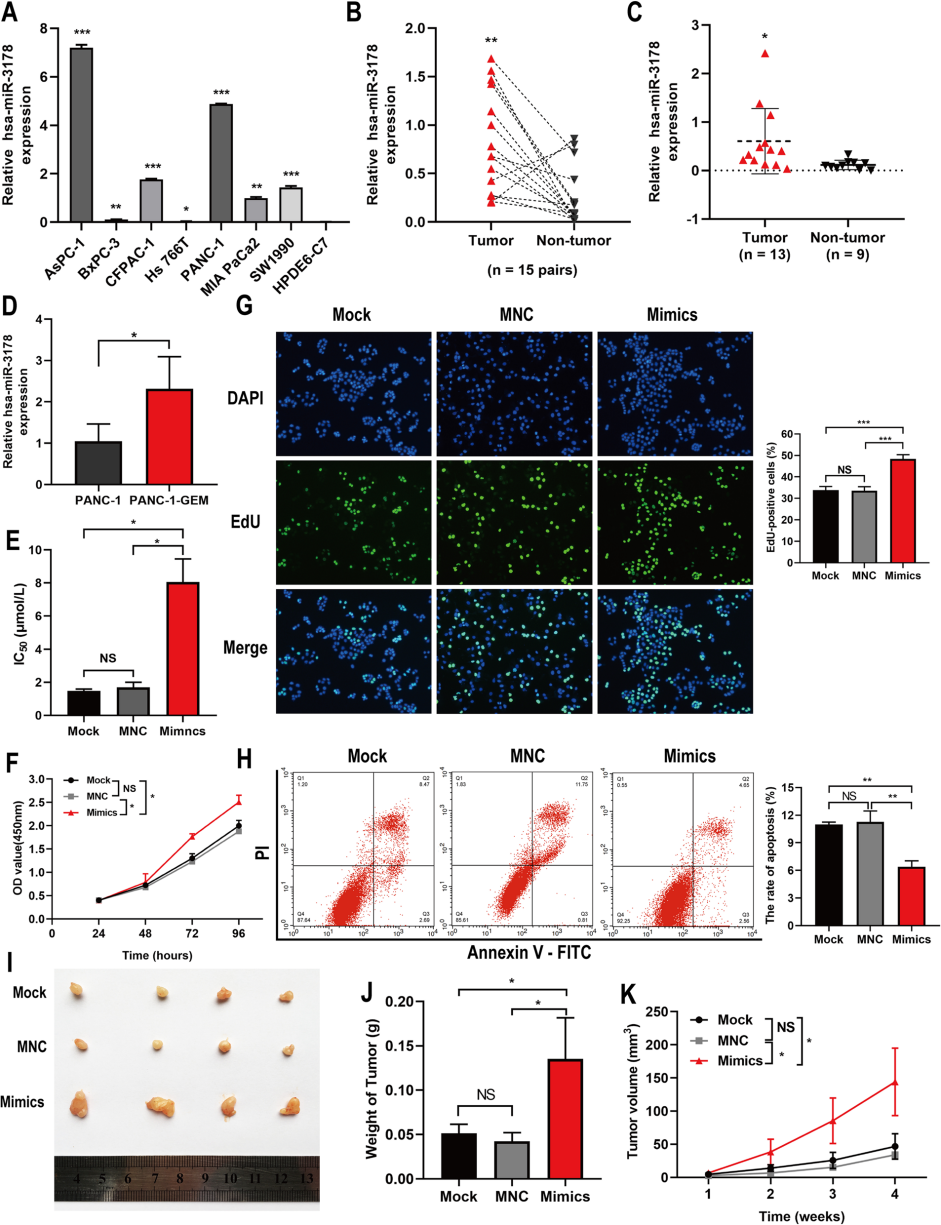
Over-expression of hsa-miR-3178 promoted proliferation of PANC-1 cells and conferred gemcitabine resistance. **A-C** qRT-PCR analysis on hsa-miR-3178 expression in seven pancreatic cancer cell lines (AsPC-1, BxPC-3, CFPAC-1, Hs766t, PANC-1, MIA PaCa2 and SW1990) and in the PC tissues compared to the primary normal human pancreatic duct epithelial cell (HPDE6-C7) or adjacent non-tumor tissues. Data are presented as mean ± SD; *P < 0.05, **P < 0.01, ***P < 0.001. **D** qRT-PCR assay for relative expression of hsa-miR-3178 in PANC-1 and PANC-1-GEM cells. **E** CCK-8 method was used to detect IC_50_ of Mock, MNC and hsa-miR-3178 mimic-transfected PANC-1 cells upon treatment with gemcitabine. **F** The CCK-8 growth curves of indicated cells upon treatment with gemcitabine (1 μmol/L). **G** The representative fluorescent micrograph and quantification on EdU staining of indicated cells upon treatment with gemcitabine (1 μmol/L). **H** Flow cytometry on cell apoptosis in indicated cells exposed to gemcitabine (1 μmol/L) for 72 h. **I** Tumor xenograft images from mice treated with gemcitabine (5 mg/kg, IP, q3d). **J** Tumor weights showed the effects of hsa-miR-3178 on the indicated groups. **K** Tumor volumes measured on the indicated times. Data are expressed as mean ± SD from three independent experiments. *P < 0.05; **P < 0.01; ***P < 0.001. PANC-1-GEM: gemcitabine resistant subline; IC_50_: half-inhibitory concentration; Mock: untreated cells; MNC: Mimic negative control; PI: Propidium Iodide; FITC: Fluorescein Isothiocyanate; IHC: immunohistochemistry; IP: intraperitoneal injection

Our previous study has shown that hsa-miR-3178 overexpression in PC is associated with poor prognosis [16]. The role of hsa-miR-3178 in gemcitabine resistance of PC was investigated. qRT-PCR analysis demonstrated the upregulation of hsa-miR-3178 in seven pancreatic cancer cell lines (AsPC-1, BxPC-3, CFPAC-1, Hs766t, PANC-1, MIA PaCa2 and SW1990) and in the PC tissues in contrast to the primary normal human pancreatic duct epithelial cell (HPDE6-C7) or adjacent non-tumor tissues (Figs. 1 A-C). We further found that hsa-miR-3178 was overexpressed in gemcitabine-resistant PANC-1-GEM cells compared with gemcitabine-sensitive PANC-1 cells (Fig. 2 D). As shown in Fig. 2 E, hsa-miR-3178 mimics increased half-inhibitory concentration (IC_50_) of gemcitabine (8.06 ± 0.81 μmol/L) compared to control groups (un-transfected cells, 1.49 ± 0.62 μmol/L; negative control, 1.70 ± 0.18 μmol/L) in PANC-1 cells.

The CCK-8 and EdU assays were performed to evaluate cell viability and proliferation. It was found that PANC-1 cells with induced hsa-miR-3178 expression showed significant resistance to gemcitabine (1 μmol/L), as manifested by increased cell proliferation (Fig. 2 F, G). We further performed flow cytometry assay to evaluate whether hsa-miR-3178 was capable of inhibiting gemcitabine-induced apoptosis in PANC-1 cells. It was found that the percentage of cell apoptosis in hsa-miR-3178 transfection group was much lower than that in the control cells (Fig. 2 H).

To further verify the above results, we generated xenograft tumor models via injecting BALB/c nude mice with PANC-1 cells (with transfection of agomir-3178, agomir negative control or PBS). As expected, hsa-miR-3178 significantly enhanced xenograft tumor growth and tumor weight at the end of the experiment even with gemcitabine treatment (Fig. 2I-K). Furthermore, hsa-miR-3178 overexpression reduced gemcitabine-induced cell apoptosis, and the TUNEL^+^ cell proportion decreased compared with the control group (Figure S1 A).

### *Down-regulation of hsa-miR-3178 inhibited PANC-1-GEM cells proliferation and increased cell sensitivity to gemcitabine both *in vitro* and *in vivo

**Fig. 3:**
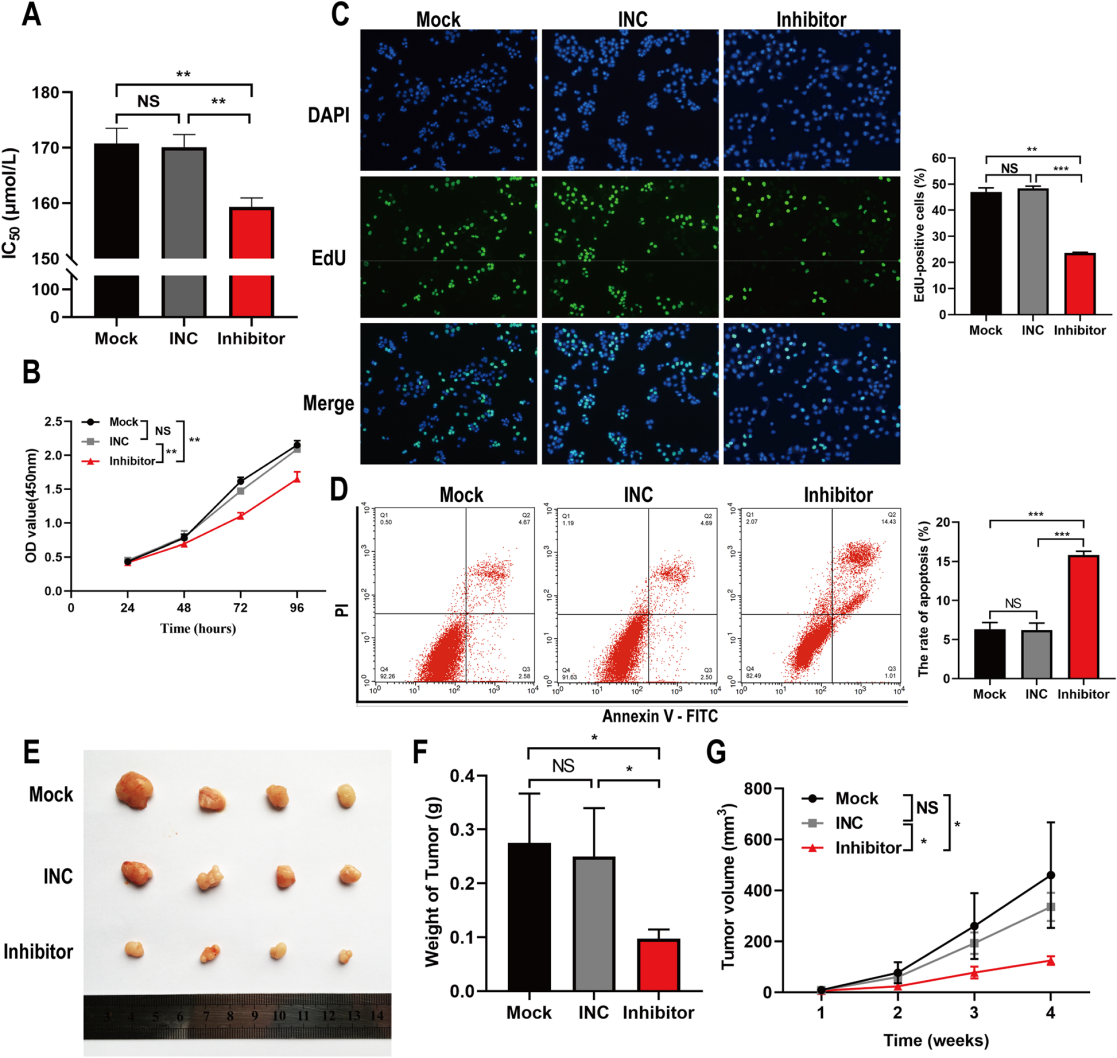
Down-regulation of hsa-miR-3178 inhibited proliferation of PANC-1-GEM cells and increased sensitivity of cells to gemcitabine. **A** CCK-8 assay on cell viability of PANC-1-GEM cells transfected with Mock, INC, and hsa-miR-3178 inhibitor and treated with gemcitabine for calculating IC_50_. **B** The CCK-8 growth curves of indicated cells upon treatment with gemcitabine (150 μmol/L). **(C)** The representative fluorescent micrograph and quantification of EdU staining on indicated cells upon treatment with gemcitabine (150 μmol/L). **D** Flow cytometry on cell apoptosis in indicated cells exposed to gemcitabine (150 μmol/L) for 72 h. **E** Tumor xenograft images from mice treated with gemcitabine (5 mg/kg, IP, q3d). **F** Tumor weights showed the effects of hsa-miR-3178 inhibitor on the indicated groups. **G** Tumor volumes measured on the indicated times. Data are expressed as mean ± SD from three independent experiments. *P < 0.05; **P < 0.01; ***P < 0.001. PANC-1-GEM: gemcitabine resistant subline. IC_50_: half-inhibitory concentration; Mock: untreated cells; INC: Inhibitor negative control; PI: Propidium Iodide; FITC: Fluorescein Isothiocyanate; IP: intraperitoneal injection

To investigate the role of hsa-miR-3178 in in drug resistance in vitro, PANC-1-GEM cells with hsa-miR-3178 silencing were established by transfecting hsa-miR-3178 inhibiting vectors. As shown in Fig. 3 A, transfection of hsa-miR-3178 inhibiting vectors decreased IC_50_ of gemcitabine (159.33 ± 0.94 μmol/L) compared to control groups (un-transfected cells, 170.77 ± 1.60 μmol/L; negative control, 170.13 ± 1.30 μmol/L) in PANC-1-GEM cells. CCK-8 and EdU assays demonstrated that PANC-1-GEM cells with reduced hsa-miR-3178 expression increased sensitivity to gemcitabine, as manifested by a significant decrease in cell proliferation (Fig. 3 B, C). Flow cytometry assays revealed that the percentage of cells apoptosis in hsa-miR-3178 inhibitor transfection group was significantly higher than that in the control cells which was the opposite of results of hsa-miR-3178 mimics transfection in PANC-1 cells (Fig. 3 D).

To confirm the above results in vivo, we generated xenograft tumor models via injecting PANC-1-GEM cells (with transfection of antagomir-3178, antagomir negative control or PBS, respectively) into BALB/c nude mice. As indicated, antagomir-3178 significantly reduced xenograft tumor weight at the end of the experiment and slowed down tumor growth following gemcitabine treatment (Fig. 3 E–G). Moreover, hsa-miR-3178 downregulation enhanced the effects of chemotherapy in vivo, and the TUNEL^+^ cell proportion increased compared with the control group (Figure S1 B).

### RhoB was a direct target gene of hsa-miR-3178

**Fig. 4:**
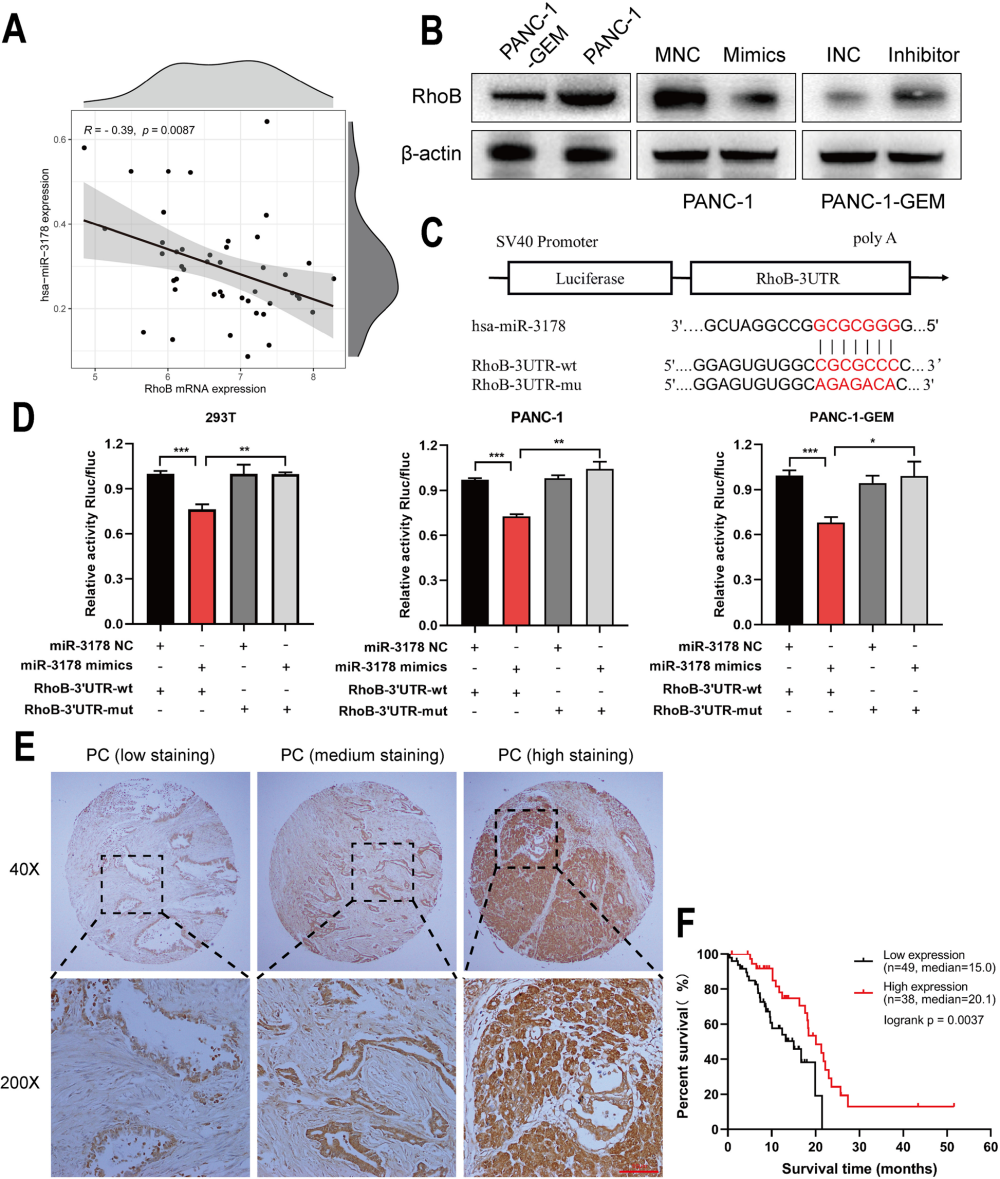
RhoB was a direct target gene of hsa-miR-3178. **A** RhoB was negatively correlated with hsa-miR-3178 expression in TCGA PC patients. **B** Western blotting on the expression of RhoB on PANC-1 cells with hsa-miR-3178 mimics and PANC-1-GEM cells with hsa-miR-3178 inhibitor, and the expression of RhoB in PANC-1, PANC-1-GEM cells. **C** Illustration of putative binding sites of hsa-miR-3178 with the 3’-UTR of RhoB. **D** Luciferase assay showed hsa-miR-3178 targeted the 3’-UTR regions of RhoB. **E–F** IHC staining of RhoB and Kaplan–Meier analysis (*n* = 87; Log-rank test; *P* = 0.0037). Data are expressed as mean ± SD from three independent experiments. **P < 0.01; ***P < 0.001

By using four bioinformatics algorithms including miRWalk, miRanda, RNA22 and Targescan, and then intersecting with down-regulated genes in PANC-1-GEM cells from gemcitabine resistance dataset GSE80617 [16], we obtained two genes RhoB and HIST2H2BE. Through literature investigation, we found that RhoB plays a biological function as a cancer promoter in pancreatic cancer [21]. To explore the regulatory relationship between RhoB and hsa-miR-3178, we probed the TCGA database contained RhoB and hsa-miR-3178 expression profiles in PC patients. As shown in Fig. 4 A, we found that the expression of RhoB was negatively correlated with hsa-miR-3178.

Moreover, hsa-miR-3178 mimics was introduced into PANC-1 cells and hsa-miR-3178 inhibitor was introduced into PANC-1-GEM cells. Western blotting revealed that upregulation of hsa-miR-3178 inhibited RhoB expression while downregulation of hsa-miR-3178 enhanced RhoB expression (Fig. 4 B).

A dual-luciferase reporter assay was performed to validate RhoB as a direct target gene of hsa-miR-3178. To do this, hsa-miR-3178 mimics, mimics NC, and plasmids containing the wild-type 3’-UTR and mutant 3’-UTR were each constructed, and the characteristics of these vectors are presented in Fig. 4 C and supplementary-word1. The results indicated that co-transfection of hsa-miR-3178 mimics and wild-type plasmids significantly decreased luciferase activity compared with that with the transfection of mutant plasmids (Fig. 4 D). These results suggested that hsa-miR-3178 may restrain RhoB expression by directly targeting its 3’-UTR.

RhoB was reported as a cancer suppressor and loss of RhoB was strongly associated with poor survival of patients [34]. However, the effect of RhoB on survival in PC has not been studied. We performed IHC assay to explore the clinical significance of RhoB expression using a tissue microarray which contains 87 cases of PC. Kaplan–Meier survival analysis revealed that overexpression of RhoB was significantly associated with a good overall survival of PC patients (Fig. 4 E–F).

### RhoB reversed hsa-miR-3178-mediated gemcitabine resistance in PC cells gemcitabine-resistant PC cells and the parental PANC-1 cells

**Fig. 5:**
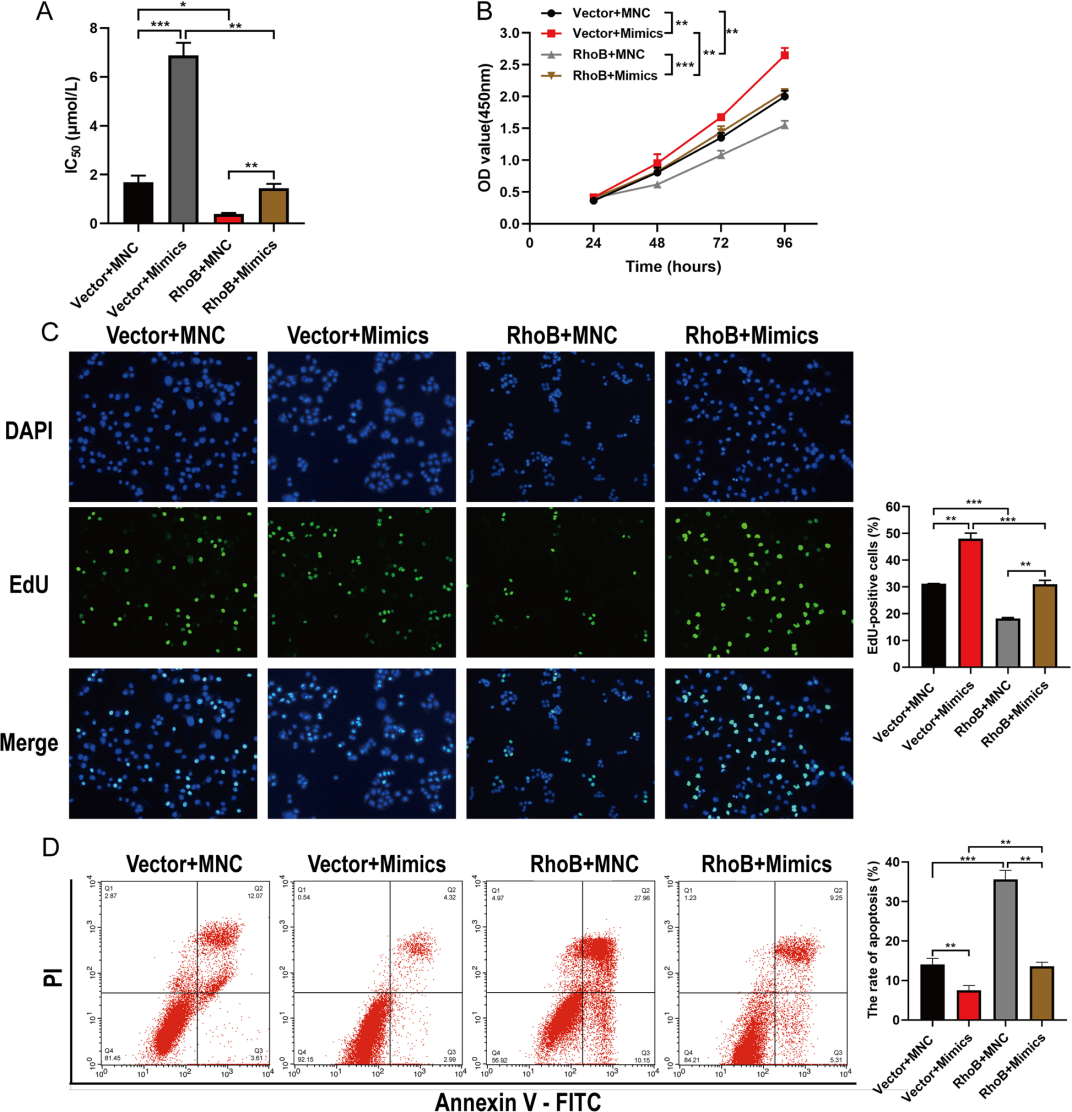
RhoB reversed hsa-miR-3178-mediated proliferation and gemcitabine resistance in PANC-1 cells. Co-transfection of RhoB overexpression lentivirus with hsa-miR-3178 mimics in PANC-1 cells. **A** CCK-8 assay for IC_50_ of gemcitabine in PANC-1 cells with indicated transfection. **B** The CCK-8 growth curves of indicated cells upon treatment with gemcitabine (1 μmol/L). **C** The representative fluorescent micrograph and quantification of the EdU staining of indicated cells upon treatment with gemcitabine (1 μmol/L). **D** Flow cytometry on cell apoptosis in indicated cells exposed to gemcitabine (1 μmol/L) for 72 h. Data are expressed as mean ± SD from three independent experiments. *P < 0.05; **P < 0.01; ***P < 0.001. IC_50_: half-inhibitory concentration; Mock: untreated cells; MNC: Mimic negative control; vector: vector-only control; PI: Propidium Iodide; FITC: Fluorescein Isothiocyanate

**Fig. 6:**
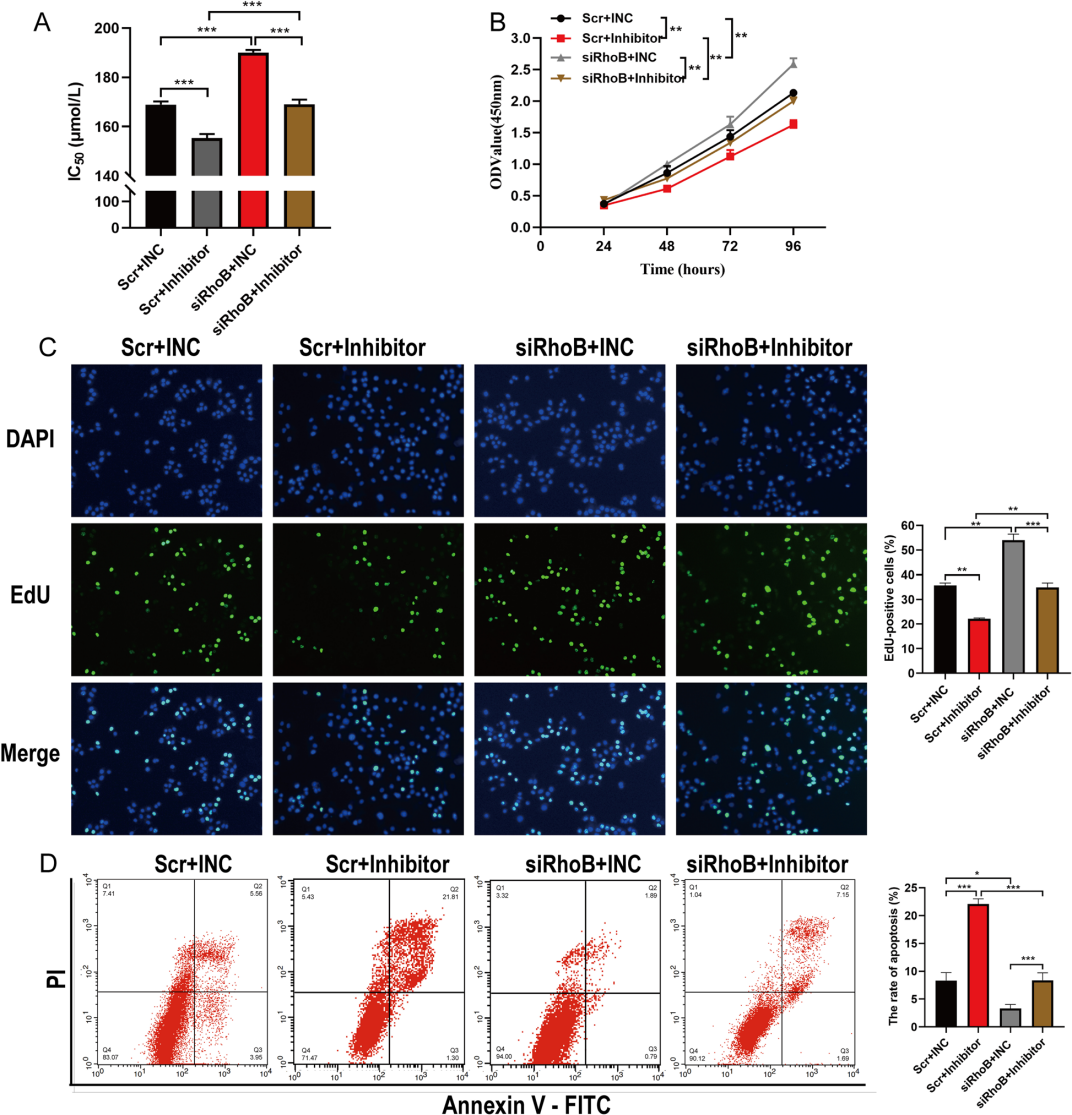
RhoB reversed hsa-miR-3178-mediated proliferation and gemcitabine resistance in PANC-1-GEM cells. Co-transfection of siRhoB with hsa-miR-3178 inhibitor in PANC-1-GEM cells. **A** CCK-8 assay for IC_50_ of gemcitabine in PANC-1-GEM cells with indicated transfection. **B** The CCK-8 growth curves of indicated cells upon treatment with gemcitabine (150 μmol/L). **C** The representative fluorescent micrograph and quantification of the EdU staining of indicated cells upon treatment with gemcitabine (150 μmol/L). **D** Flow cytometry on cell apoptosis in indicated cells exposed to gemcitabine (150 μmol/L) for 72 h. Data are expressed as mean ± SD from three independent experiments. *P < 0.05; **P < 0.01; ***P < 0.001. IC_50_: half-inhibitory concentration; Mock: untreated cells; INC: Inhibitor negative control; vector: vector-only control; siRhoB: RhoB small interfering RNA; Scr: scramble control; PI: Propidium Iodide; FITC: Fluorescein Isothiocyanate

To further confirm the role of RhoB suppression by hsa-miR-3178 in PC cell proliferation and gemcitabine resistance, we co-transfected RhoB overexpression lentivirus with hsa-miR-3178 mimics in PANC-1 cells or RhoB small interfering RNA (siRNA) with hsa-miR-3178 inhibitor in PANC-1-GEM cells. As shown in Fig. 5 A, upregulation of RhoB decreased the IC_50_ of PANC-1 cells to gemcitabine (Vector + MNC, 1.68 ± 0.16 μmol/L; RhoB + MNC, 0.39 ± 0.02 μmol/L) and reversed hsa-miR-3178-mediated gemcitabine resistance in PANC-1 cells (Vector + Mimics, 6.87 ± 0.30 μmol/L; RhoB + Mimics, 1.44 ± 0.10 μmol/L). On the contrary, downregulation of RhoB increased the IC_50_ of PANC-1-GEM cells to gemcitabine (Scr + INC, 168.93 ± 0.75 μmol/L; siRhoB + INC, 190.07 ± 0.60 μmol/L) and antagonized hsa-miR-3178 inhibitor-mediated gemcitabine re-sensitization in PANC-1-GEM cells (Scr + Inhibitor, 155.40 ± 0.93 μmol/L; siRhoB + Inhibitor, 169.10 ± 1.07 μmol/L) (Fig. 6 A). Furthermore, CCK-8 and EdU assay results revealed that RhoB overexpression reduced proliferation of PANC-1 cells and reversed the promotion of cell proliferation by hsa-miR-3178 in PANC-1 cells (Fig. 5 B, C). However, RhoB knockdown induced proliferation of PANC-1-GEM cells and antagonized the inhibition of cell proliferation by hsa-miR-3178 inhibitor in PANC-1-GEM cells (Fig. 6 B, C).

In addition, flow cytometric analysis revealed that overexpression of RhoB promoted apoptosis of PANC-1 cells and also antagonized the suppression of cell apoptosis by hsa-miR-3178 in PANC-1 cells (Fig. 5 D). On the contrary, RhoB knockdown suppressed cell apoptotic rate compared with control group and reversed the increased apoptosis by hsa-miR-3178 inhibitor in PANC-1-GEM cells (Fig. 6 D). Taken together, these results demonstrated that RhoB could reverse hsa-miR-3178-mediated proliferation and gemcitabine resistance in PC cells.

### Hsa-miR-3178/RhoB axis regulated gemcitabine resistance by PI3K/Akt signaling pathway and ABC transporters

Previous studies have reported that RhoB negatively regulates the PI3K/Akt signaling pathway [20, 35]. As shown in figure S1 A-B, IHC assay revealed that hsa-miR-3178 promoted expression of p-PI3K and p-Akt 473 in vivo. To explore the association of hsa-miR-3178, RhoB and PI3K/Akt signaling pathway, we performed Western blotting analyses to probe the protein expression involved in PI3K/Akt signaling pathway in PANC-1 cells and PANC-1-GEM cells with indicated treatments.

**Fig. 7:**
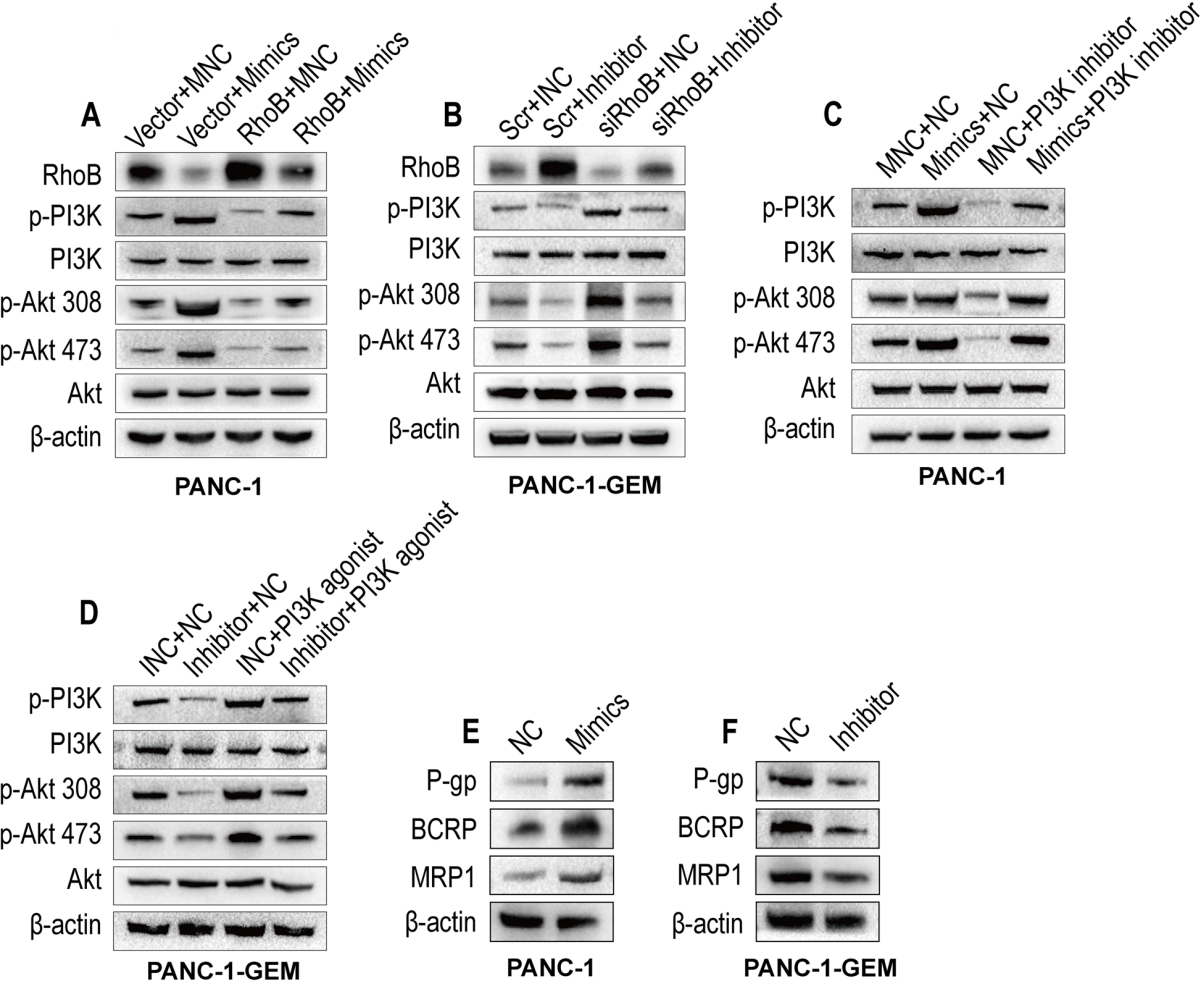
Hsa-miR-3178/RhoB axis mediated tumor growth and gemcitabine resistance by PI3K/Akt signaling pathway in PC cells. **A-F** Western blotting on the expression of RhoB, phosphorylated-PI3K, total PI3K, phosphorylated-Akt 308, phosphorylated-Akt 473, total Akt and P-gp, BCRP, MRP1 in the indicated cells. β-actin was used as a loading control. IC_50_: half-inhibitory concentration

As shown in Fig. 7 A, RhoB overexpression decreased expression of p-PI3K, p-Akt 308 and p-Akt 473, while total PI3K and total Akt expression was not affected in PANC-1 cells. Co-transfection of RhoB and hsa-miR-3178 mimics reduced the expression of p-PI3K, p-Akt 308 and p-Akt 473 by hsa-miR-3178 in PANC-1 cells. On the contrary, RhoB knockdown increased the expression of p-PI3K, p-Akt 308 and p-Akt 473 and co-transfection of siRhoB and hsa-miR-3178 inhibitor reversed the downregulated expression of p-Akt 308 and p-Akt 473 by hsa-miR-3178 inhibitor in PANC-1-GEM cells (Fig. 7 B).

**Fig. 8:**
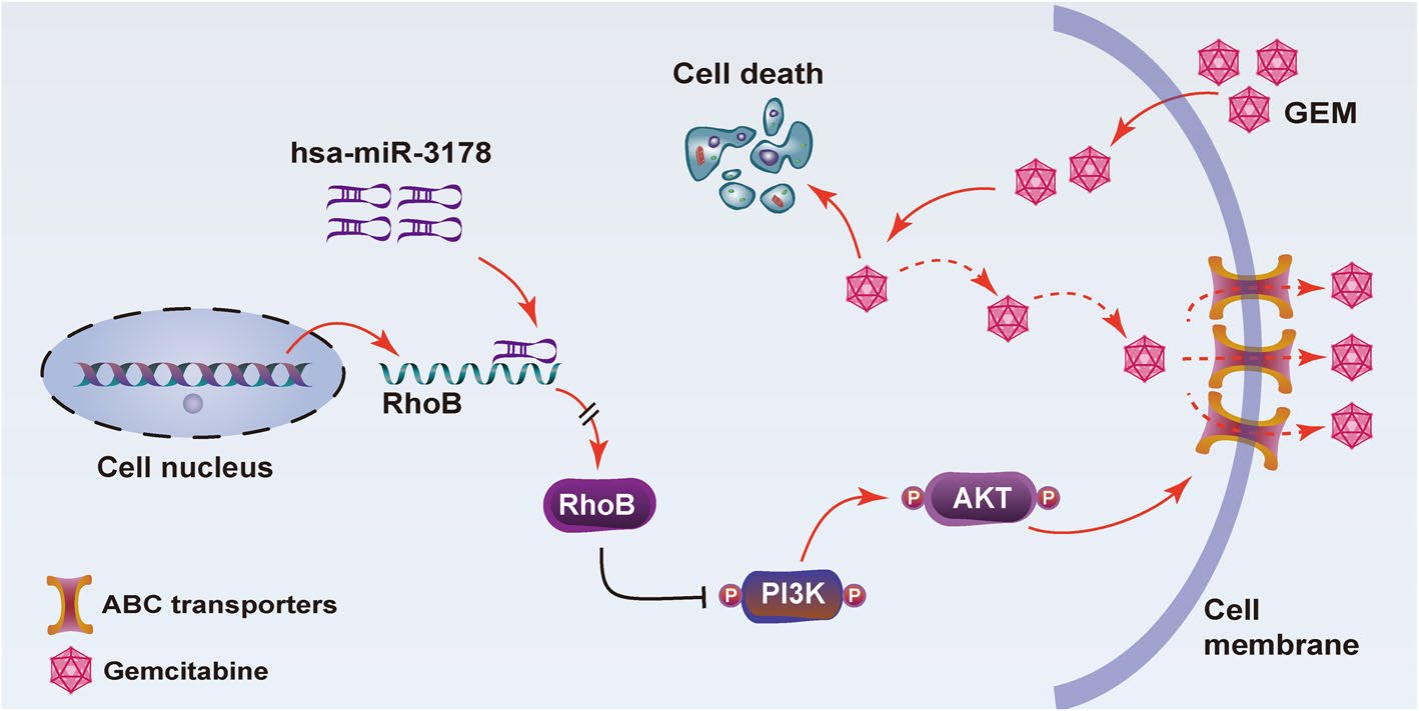
Schematic illustration of potential mechanisms of hsa-miR-3178/RhoB axis mediates tumor growth and gemcitabine resistance via PI3K/Akt pathway-mediated overexpression of ABC transporters in PC cells

To further confirm the role of PI3K/Akt signaling pathway involved in regulating proliferation and gemcitabine resistance via hsa-miR-3178/RhoB axis in PC, PANC-1 cells and PANC-1-GEM cells were incubated with the PI3K inhibitor LY294002 and PI3K agonist 740Y-P. As expected, the stimulatory effect of hsa-miR-3178 overexpression on p-PI3K, p-Akt 308 and p-Akt 473 was inhibited by PI3K inhibitor in PANC-1 cells (Fig. 7 C). On the contrary, the inhibition of downregulated hsa-miR-3178 on p-PI3K, p-Akt 308 and p-Akt 473 was reversed by PI3K agonist in PANC-1-GEM cells (Fig. 7 D). In addition, Hsa-miR-3178 overexpression in PANC-1 cells increased expression of P-gp, BCRP and MRP1 in vitro and in vivo (Fig. 7 E, Figure S1 A), whereas, hsa-miR-3178 inhibitor decreased the expression of P-gp, BCRP and MRP1 in vitro and in vivo (Fig. 7 F, Figure S1 B). To explore the regulatory relationship between PI3K/Akt signaling pathway and ABC transporters, we probed the TCGA and GTEx data contained PIK3CA, PIK3CB, PIK3CD, PIK3CG, AKT1 and the three ABC transporters expression profiles. As shown in Figure S2, we found that the expression of PI3K/Akt was positively correlated with the ABC transporters. These results suggested that hsa-miR-3178 induces gemcitabine resistance by activating PI3K/Akt signaling pathway-mediated ABC transporters in PC cells (Fig. 8).

## Discussion

The mechanism by which ABC transporters regulate gemcitabine resistance in pancreatic cancer remains unclear. In this study, we propose that hsa-miR-3178 inhibits RhoB to promote gemcitabine resistance in pancreatic cancer via activating PI3K/Akt signaling pathway and upregulating ABC transporters. We found that P-gp, BCRP and MRP1 were highly expressed in gemcitabine-resistant pancreatic cancer tissues and cells by IHC and Western blotting. Molecular docking assay demonstrated that gemcitabine had high binding affinity with P-gp, BCRP and MRP1. Previous studies suggested that miRNAs have an important regulatory relationship with ABC transporters [36]. Our previous study indicates that hsa-miR-3178 is associated with gemcitabine resistance in pancreatic cancer [16]. However, the mechanism of miR-3178 in regulating ABC transporter to induce gemcitabine resistance is not clear. In the present study, we identified overexpression of hsa-miR-3178 in gemcitabine-resistant PANC-1-GEM cells as compared to its parental PANC-1 cells. Gain- and loss-of-function studies revealed that hsa-miR-3178 induces cellular proliferation and gemcitabine resistance in PC cells. By bioinformatics analysis, Western blotting, and dual luciferase reporter assay, we predicted and confirmed that hsa-miR-3178 was a potential upstream miRNA of RhoB. And IHC assay and Kaplan–Meier survival analysis indicated that higher RhoB level was significantly associated with better overall survival of PC patients. We further investigated the important role of hsa-miR-3178/RhoB axis in regulating cellular gemcitabine resistance via PI3K/Akt signaling pathway. In vitro cell experiments and in vivo animal experiments confirmed that hsa-miR-3178 overexpression could upregulate the expression of PI3K/Akt and P-gp, BCRP and MRP1. A number of studies have confirmed that PI3K/Akt signaling pathway is related to chemotherapy resistance caused by ABC transporters [37, 38]. Our results suggested that increased hsa-miR-3178 activates PI3K/Akt signaling pathway, upregulates the expression of ABC transporters and aggravates gemcitabine resistance in pancreatic cancer. Thus, hsa-miR-3178 could be a potentially therapeutic target for PC treatment.

Previous studies have reported the roles of miRNAs on gemcitabine-based chemotherapy in various cancers [39]. In breast cancer, low expression of miR-205-5p leads to upregulation of ERp29 and decreased sensitivity of cancer cells to gemcitabine [40]. In PC, by delivering miR-210, exosomes derived from gemcitabine-resistant cancer stem cells reinforce gemcitabine resistance [41]. Our previous research found that hsa-miR-3178 was associated with gemcitabine resistance in pancreatic cancer [13]. In this study, we identified its mechanism in promoting gemcitabine resistance.

As a well-known tumor suppressor, RhoB was reported to be relevant to cisplatin resistance [18, 22, 42]. Also, previous studies have reported that RhoB was targeted by miRNAs [20, 43]. In this study, we demonstrated that hsa-miR-3178 promoted gemcitabine resistance of PC by targeting RhoB, and upregulation of RhoB could reverse hsa-miR-3178-mediated gemcitabine resistance. The regulatory networks of RhoB are very complex. Particularly, it was reported that RhoB negatively regulates the PI3K/Akt signaling pathway. In lung cancer, loss of RhoB accelerates lung cancer progression through activation of PI3K/Akt signaling pathway [35]. In osteosarcoma, miR-19 targets RhoB and down-regulates its expression, inhibiting the dephosphorylation of Akt1 protein and promoting tumor cell metastasis [20]. In line with these reports, our results demonstrated that RhoB overexpression leads to overexpression of p-PI3K, p-Akt 308 and p-Akt 473 in PANC-1 cells; whereas RhoB knockdown leads to lower their expression in PANC-1-GEM cells. The results of co-transfection with RhoB and hsa-miR-3178 and treatment with PI3K inhibitor LY294002 and PI3K agonist 740Y-P further suggested that RhoB reduces proliferation and gemcitabine resistance by antagonizing the PI3K/Akt signaling pathway in PC cells.

Research has found that overexpression of ABC drug transporters, such as P-gp, BCRP and MRP1 confers an acquired MDR due to their capabilities of transporting a broad range of chemically diverse anticancer drugs. For example, P-gp and MRP1 induced gemcitabine resistance in pancreatic cancer cells [4, 31–33]. Our previous study uncovered that PI3K inhibitor BAY-1082439 was able to reverse P-gp-mediated drug resistance in oral epidermoid carcinoma cell line and non-small cell lung cancer cell line, and the mechanism of action of BAY-1082439 is to inhibit PI3K which can downregulate the expression of P-gp and BCRP [37]. Since P-gp plays an important role in MDR, it has become an urgent task for researchers to find effective P-gp inhibitors [44, 45]. However, no positive results were obtained in clinical trials on P-gp inhibitors due to drug toxicity caused by the non-specific distribution of inhibitors and the pharmacokinetic changes caused by the interaction with a variety of anti-tumor drugs [46]. The limitation of this study is that only one resistant cell line was used for validation. Subsequent gemcitabine-resistant cell lines in pancreatic cancer need further validation of our conclusions. Another limitation is we cannot implement the biochemical experiments to prove the binding of ABC transporters to gemcitabine. Further validation was need to be performed.

## Conclusion

In conclusion, this study indicates that the hsa-miR-3178/RhoB/PI3K/Akt-mediated upregulation of ABC transporters play a vital role in inducing gemcitabine resistance in pancreatic cancer cells. Targeting hsa-miR-3178/RhoB may represent a potential therapeutic strategy to circumvent ABC transporters-mediated gemcitabine resistance for PC treatment.

## Supplementary Information

**Additional file 1: Supplementary Word 1.** h-RHOB 3-UTR reporter vector.

**Additional file 2: Supplementary Figure 1.** Hsa-miR-3178 restrainsexpression of RhoB and promotes expression of phosphorylated-PI3K andphosphorylated-Akt 473 in vivo. (A, B) IHC on theexpression of RhoB, phosphorylated-PI3K, phosphorylated-Akt 473, P-gp, BCRP andMRP1 in the indicated groups. And the TUNEL+ cell proportion in the indicatedgroups. Scale bar, 50 μm.Data are expressed as mean ±SD from three independentexperiments. **P < 0.01; ***P < 0.001. IHC: immunohistochemistry. TUNEL:Terminal-deoxynucleoitidyl Transferase Mediated Nick End Labeling.

**Additional file 3: Supplementary Figure 2. **PI3K/Aktwere positively correlated with the three ABC transporters in TCGA and GTExdatabases.

## Data Availability

Not applicable.

## Abbreviations

PC: Pancreatic Cancer
miRNAs: MicroRNAs
RhoB: Ras Homolog Family Member B
PANC-1-GEM: Gemcitabine-resistant PANC-1 cell
ABC transporters: ATP-Binding Cassette Transporters
P-gp: P-glycoprotein
BCRP: Breast Cancer Resistance Protein
MRP1: Multidrug Resistance Protein 1
INC: Inhibitor Negative Control
MNC: Mimic Negative Control
Scr: Scramble Control
IHC: Immunohistochemistry
CCK-8: Cell Counting Kit 8
EdU: 5-Ethynyl-2’-deoxyuridine
HE: Hematoxylin–Eosin Staining
TUNEL: Terminal deoxynucleotidyl transferase (TdT) dUTP nick-end labeling

## References

[1] Siegel RL, Miller KD, Fuchs HE, Jemal A (2021). Cancer Statistics, 2021. CA Cancer J Clin.

[2] Kamisawa T, Wood LD, Itoi T, Takaori K (2016). Pancreatic cancer. Lancet.

[3] Binenbaum Y, Na'ara S, Gil Z (2015). Gemcitabine resistance in pancreatic ductal adenocarcinoma. Drug Resist Updat.

[4] Ji N, Yang Y, Cai CY, Lei ZN, Wang JQ, Gupta P, Teng QX, Chen ZS, Kong D, Yang DH (2018). VS-4718 Antagonizes Multidrug Resistance in ABCB1- and ABCG2-Overexpressing Cancer Cells by Inhibiting the Efflux Function of ABC Transporters. Front Pharmacol.

[5] Muriithi W, Macharia LW, Heming CP, Echevarria JL, Nyachieo A, Filho PN, Neto VM (2020). ABC transporters and the hallmarks of cancer: roles in cancer aggressiveness beyond multidrug resistance. Cancer Biol Med.

[6] Migliore C, Giordano S (2013). Resistance to targeted therapies: a role for microRNAs?. Trends Mol Med.

[7] Garofalo M, Croce CM (2013). MicroRNAs as therapeutic targets in chemoresistance. Drug Resist Updat.

[8] MadurantakamRoyam M, Ramesh R, Shanker R, Sabarimurugan S, Kumarasamy C, Ramesh N, Gothandam KM, Baxi S, Gupta A, Krishnan S, Jayaraj R (2019). miRNA Predictors of Pancreatic Cancer Chemotherapeutic Response: A Systematic Review and Meta-Analysis. Cancers (Basel).

[9] Bartel DP (2004). MicroRNAs: genomics, biogenesis, mechanism, and function. Cell.

[10] Jain CK, Gupta A, Dogra N, Kumar VS, Wadhwa G, Sharma SK (2014). MicroRNA therapeutics: the emerging anticancer strategies. Recent Pat Anticancer Drug Discov.

[11] Cui M, Yao X, Lin Y, Zhang D, Cui R, Zhang X (2020). Interactive functions of microRNAs in the miR-23a-27a-24-2 cluster and the potential for targeted therapy in cancer. J Cell Physiol.

[12] Mahmoudian-Sani MR, Asadi-Samani M (2020). Modulation of MicroRNAs by Euphorbia Microsciadia Boiss in MDA-MB-231 Cell Line: New Possibilities in Breast Cancer Therapy. Recent Pat Anticancer Drug Discov.

[13] Kong P, Chen L, Yu M, Tao J, Liu J, Wang Y, Pan H, Zhou W, Wang S (2018). miR-3178 inhibits cell proliferation and metastasis by targeting Notch1 in triple-negative breast cancer. Cell Death Dis.

[14] Wang T, Chen G, Ma X, Yang Y, Chen Y, Peng Y, Bai Z, Zhang Z, Pei H, Guo W (2019). MiR-30a regulates cancer cell response to chemotherapy through SNAI1/IRS1/AKT pathway. Cell Death Dis.

[15] Funamizu N, Lacy CR, Kamada M, Yanaga K, Manome Y (2019). MicroRNA-200b and -301 are associated with gemcitabine response as biomarkers in pancreatic carcinoma cells. Int J Oncol.

[16] Gu J, Zhang J, Huang W, Tao T, Huang Y, Yang L, Yang J, Fan Y, Wang H (2020). Activating miRNA-mRNA network in gemcitabine-resistant pancreatic cancer cell associates with alteration of memory CD4(+) T cells. Ann Transl Med.

[17] Sahai E, Marshall CJ (2002). RHO-GTPases and cancer. Nat Rev Cancer.

[18] Mokady D, Meiri D (2015). RhoGTPases - A novel link between cytoskeleton organization and cisplatin resistance. Drug Resist Updat.

[19] Schaefer A, Reinhard NR, Hordijk PL (2014). Toward understanding RhoGTPase specificity: structure, function and local activation. Small GTPases.

[20] Zou Q, Xiao X, Liang Y, Peng L, Guo Z, Li W, Yu W (2018). miR-19a-mediated downregulation of RhoB inhibits the dephosphorylation of AKT1 and induces osteosarcoma cell metastasis. Cancer Lett.

[21] Tan Y, Yin H, Zhang H, Fang J, Zheng W, Li D, Li Y, Cao W, Sun C, Liang Y (2015). Sp1-driven up-regulation of miR-19a decreases RHOB and promotes pancreatic cancer. Oncotarget.

[22] Cimbora-Zovko T, Fritz G, Mikac N, Osmak M (2010). Downregulation of RhoB GTPase confers resistance to cisplatin in human laryngeal carcinoma cells. Cancer Lett.

[23] Huang GX, Pan XY, Jin YD, Wang Y, Song XL, Wang CH, Li YD, Lu J (2016). The mechanisms and significance of up-regulation of RhoB expression by hypoxia and glucocorticoid in rat lung and A549 cells. J Cell Mol Med.

[24] Li J, Wu H, Li W, Yin L, Guo S, Xu X, Ouyang Y, Zhao Z, Liu S, Tian Y (2016). Downregulated miR-506 expression facilitates pancreatic cancer progression and chemoresistance via SPHK1/Akt/NF-κB signaling. Oncogene.

[25] Li J, Wu H, Li W, Yin L, Guo S, Xu X, Ouyang Y, Zhao Z, Liu S, Tian Y (2016). Downregulated miR-506 expression facilitates pancreatic cancer progression and chemoresistance via SPHK1/Akt/NF-kappaB signaling. Oncogene.

[26] Alam A, Küng R, Kowal J, McLeod RA, Tremp N, Broude EV, Roninson IB, Stahlberg H, Locher KP (2018). Structure of a zosuquidar and UIC2-bound human-mouse chimeric ABCB1. Proc Natl Acad Sci U S A.

[27] Jackson SM, Manolaridis I, Kowal J, Zechner M, Taylor NMI, Bause M, Bauer S, Bartholomaeus R, Bernhardt G, Koenig B (2018). Structural basis of small-molecule inhibition of human multidrug transporter ABCG2. Nat Struct Mol Biol.

[28] Johnson ZL, Chen J (2017). Structural Basis of Substrate Recognition by the Multidrug Resistance Protein MRP1. Cell.

[29] Cai CY, Zhang W, Wang JQ, Lei ZN, Zhang YK, Wang YJ, Gupta P, Tan CP, Wang B, Chen ZS (2020). Biological evaluation of non-basic chalcone CYB-2 as a dual ABCG2/ABCB1 inhibitor. Biochem Pharmacol.

[30] Ji N, Yang Y, Cai CY, Lei ZN, Wang JQ, Gupta P, Shukla S, Ambudkar SV, Kong D, Chen ZS (2019). Selonsertib (GS-4997), an ASK1 inhibitor, antagonizes multidrug resistance in ABCB1- and ABCG2-overexpressing cancer cells. Cancer Lett.

[31] Kohan HG, Boroujerdi M (2015). Time and concentration dependency of P-gp, MRP1 and MRP5 induction in response to gemcitabine uptake in Capan-2 pancreatic cancer cells. Xenobiotica.

[32] El-Mahdy HA, El-Husseiny AA, Kandil YI, Gamal El-Din AM (2020). Diltiazem potentiates the cytotoxicity of gemcitabine and 5-fluorouracil in PANC-1 human pancreatic cancer cells through inhibition of P-glycoprotein. Life Sci.

[33] Chang XB (2007). A molecular understanding of ATP-dependent solute transport by multidrug resistance-associated protein MRP1. Cancer Metastasis Rev.

[34] Calvayrac O, Pradines A, Raymond-Letron I, Rouquette I, Bousquet E, Lauwers-Cances V, Filleron T, Cadranel J, Beau-Faller M, Casanova A (2014). RhoB determines tumor aggressiveness in a murine EGFRL858R-induced adenocarcinoma model and is a potential prognostic biomarker for Lepidic lung cancer. Clin Cancer Res.

[35] Bousquet E, Mazieres J, Privat M, Rizzati V, Casanova A, Ledoux A, Mery E, Couderc B, Favre G, Pradines A (2009). Loss of RhoB expression promotes migration and invasion of human bronchial cells via activation of AKT1. Cancer Res.

[36] Wang Y, Wang Y, Qin Z, Cai S, Yu L, Hu H, Zeng S (2021). The role of non-coding RNAs in ABC transporters regulation and their clinical implications of multidrug resistance in cancer. Expert Opin Drug Metab Toxicol..

[37] Zhang L, Li Y, Wang Q, Chen Z, Li X, Wu Z, Hu C, Liao D, Zhang W, Chen ZS (2020). The PI3K subunits, P110α and P110β are potential targets for overcoming P-gp and BCRP-mediated MDR in cancer. Mol Cancer.

[38] Zhao L, Zhang W, Zhang F (2021). Poncirin downregulates ATP-binding cassette transporters to enhance cisplatin sensitivity in cisplatin-resistant osteosarcoma cells. Phytother Res.

[39] Assaraf YG, Brozovic A, Gonçalves AC, Jurkovicova D, Linē A, Machuqueiro M, Saponara S, Sarmento-Ribeiro AB, Xavier CPR, Vasconcelos MH (2019). The multi-factorial nature of clinical multidrug resistance in cancer. Drug Resist Updat.

[40] Ma C, Shi X, Guo W, Feng F, Wang G (2019). miR-205-5p downregulation decreases gemcitabine sensitivity of breast cancer cells via ERp29 upregulation. Exp Ther Med.

[41] Yang Z, Zhao N, Cui J, Wu H, Xiong J, Peng T (2020). Exosomes derived from cancer stem cells of gemcitabine-resistant pancreatic cancer cells enhance drug resistance by delivering miR-210. Cell Oncol (Dordr).

[42] Liu N, Cui W, Jiang X, Zhang Z, Gnosa S, Ali Z, Jensen L, Jonsson JI, Blockhuys S, Lam EW (2019). The Critical Role of Dysregulated RhoB Signaling Pathway in Radioresistance of Colorectal Cancer. Int J Radiat Oncol Biol Phys.

[43] Wei LJ, Li JA, Bai DM, Song Y (2018). miR-223-RhoB signaling pathway regulates the proliferation and apoptosis of colon adenocarcinoma. Chem Biol Interact.

[44] Zhang H, Xu H, Ashby CR, Assaraf YG, Chen ZS, Liu HM (2021). Chemical molecular-based approach to overcome multidrug resistance in cancer by targeting P-glycoprotein (P-gp). Med Res Rev..

[45] Dong J, Qin Z, Zhang WD, Cheng G, Yehuda AG, Ashby CR, Chen ZS, Cheng XD, Qin JJ (2020). Medicinal chemistry strategies to discover P-glycoprotein inhibitors: An update. Drug Resist Updat.

[46] Kathawala RJ, Gupta P, Ashby CR, Chen ZS (2015). The modulation of ABC transporter-mediated multidrug resistance in cancer: a review of the past decade. Drug Resist Updat.

