# Supplementary material for: Hsa-miR-3178/RhoB/PI3K/Akt, a novel signaling pathway regulates ABC transporters to reverse gemcitabine resistance in pancreatic cancer
Source: Mol Cancer. 2022 May 10;21:112. doi: 10.1186/s12943-022-01587-9 (PMC9088115; doi:10.1186/s12943-022-01587-9)
Supplement: Supplementary file 1 — Additional file 1: Supplementary Word 1. h-RHOB 3-UTR reporter vector. [file 12943_2022_1587_MOESM1_ESM.docx]

**h-RHOB** **3-UTR reporter vector**

1. Vector and target gene information

1) pSI-Check2 Vector map:


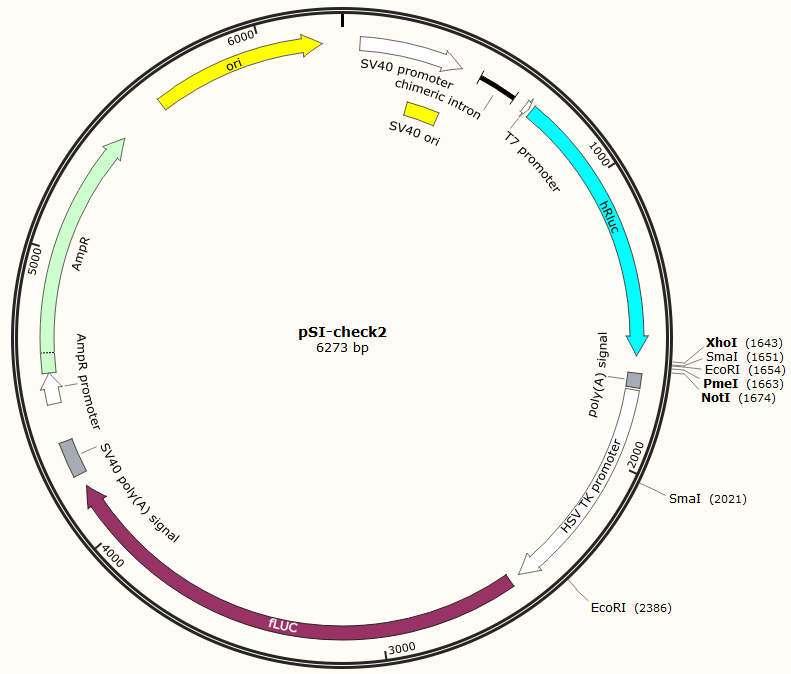


2. h-RHOB sequence：

h-RHOB-3UTR-wt:
GGGCCGCGCCCGTCGCGCCTGCCCCTGCCGGCACGGCTCCCCCTCCTGGACCAGTCCCCCGCGAGCCCGGAGAAGGGGAGACCCGTGTCCCACAAGGACCCCACCGGCCTGCCTGGCATCTGTCTGCTGACGCCTCTGGCTTGCGCCAGGACTTGGCGTGGGCACCGGGCGCCCCCATCCCAGTGTCTGTGTGCGTCCAGCTGTGTTGCACAGGCCTGGGCTCCCCACTGAGTGCCAAGGGTCCCCTGAGCATGCTTTTCTGAAGAGCCGGGCCTCAGAGTGTGTGGCTGTGTGTCTGTTCGACTCCCCTCGCCCCATTTTCACCCCACCCCCGCCTCTGATCCCCGGGGGCGAGATTGGCGCGGGAGTGTGGCCGCGCCCCATCAGATGTTCGCCCTTCACCAGCGGGAGCTTGATATCCCTTGTCTGTAACATAGACCCCGGGTACTGCGGGAGGGGAGGGCTGCTGGGGAGGATGGGGGGATGTTATATAAATATAG

h-RHOB-3UTR-mu:
GGGCaGaGaCaGTaGaGaCTGCCCCTGCCGGCACGGCTCCCCCTCCTGGACCAGTCCCCCGCGAGCCCGGAGAAGGGGAGACCCGTGTCCCACAAGGACCCCACCGGCCTGCCTGGCATCTGTCTGCTGACGCCTCTGGCTTGCGCCAGGACTTGGCGTGGGCACCGGGCGCCCCCATCCCAGTGTCTGTGTGCGTCCAGCTGTGTTGCACAGGCCTGGGCTCCCCACTGAGTGCCAAGGGTCCCCTGAGCATGCTTTTCTGAAGAGCCGGGCCTCAGAGTGTGTGGCTGTGTGTCTGTTCGACTCCCCTCGCCCCATTTTCACCCCACCCCCGCCTCTGATCCCCGGGGGCGAGATTGGCGCGGGAGTGTGGCaGaGaCaCATCAGATGTTCGCCCTTCACCAGCGGGAGCTTGATATCCCTTGTCTGTAACATAGACCCCGGGTACTGCGGGAGGGGAGGGCTGCTGGGGAGGATGGGGGGATGTTATATAAATATAG
>hsa-miR-3178 MIMAT0015055
GGGGCGCGGCCGGAUCG

3. h-RHOB Vector sequencing results:

h-RHOB-3UTR-wt:

ctcgagGGGCCGCGCCCGTCGCGCCTGCCCCTGCCGGCACGGCTCCCCCTCCTGGACCAGTCCCCCGCGAGCCCGGAGAAGGGGAGACCCGTGTCCCACAAGGACCCCACCGGCCTGCCTGGCATCTGTCTGCTGACGCCTCTGGCTTGCGCCAGGACTTGGCGTGGGCACCGGGCGCCCCCATCCCAGTGTCTGTGTGCGTCCAGCTGTGTTGCACAGGCCTGGGCTCCCCACTGAGTGCCAAGGGTCCCCTGAGCATGCTTTTCTGAAGAGCCGGGCCTCAGAGTGTGTGGCTGTGTGTCTGTTCGACTCCCCTCGCCCCATTTTCACCCCACCCCCGCCTCTGATCCCCGGGGGCGAGATTGGCGCGGGAGTGTGGC**CGCGCCC**CATCAGATGTTCGCCCTTCACCAGCGGGAGCTTGATATCCCTTGTCTGTAACATAGACCCCGGGTACTGCGGGAGGGGAGGGCTGCTGGGGAGGATGGGGGGATGTTATATAAATATAGgcggccgc

h-RHOB-3UTR-mu:

ctcgagGGGCaGaGaCaGTaGaGaCTGCCCCTGCCGGCACGGCTCCCCCTCCTGGACCAGTCCCCCGCGAGCCCGGAGAAGGGGAGACCCGTGTCCCACAAGGACCCCACCGGCCTGCCTGGCATCTGTCTGCTGACGCCTCTGGCTTGCGCCAGGACTTGGCGTGGGCACCGGGCGCCCCCATCCCAGTGTCTGTGTGCGTCCAGCTGTGTTGCACAGGCCTGGGCTCCCCACTGAGTGCCAAGGGTCCCCTGAGCATGCTTTTCTGAAGAGCCGGGCCTCAGAGTGTGTGGCTGTGTGTCTGTTCGACTCCCCTCGCCCCATTTTCACCCCACCCCCGCCTCTGATCCCCGGGGGCGAGATTGGCGCGGGAGTGTGGC**aGaGaCa**CATCAGATGTTCGCCCTTCACCAGCGGGAGCTTGATATCCCTTGTCTGTAACATAGACCCCGGGTACTGCGGGAGGGGAGGGCTGCTGGGGAGGATGGGGGGATGTTATATAAATATAGgcggccgc
