# Supplementary figures and images for: Hsa-miR-3178/RhoB/PI3K/Akt, a novel signaling pathway regulates ABC transporters to reverse gemcitabine resistance in pancreatic cancer
Source: Mol Cancer. 2022 May 10;21:112. doi: 10.1186/s12943-022-01587-9 (PMC9088115; doi:10.1186/s12943-022-01587-9)

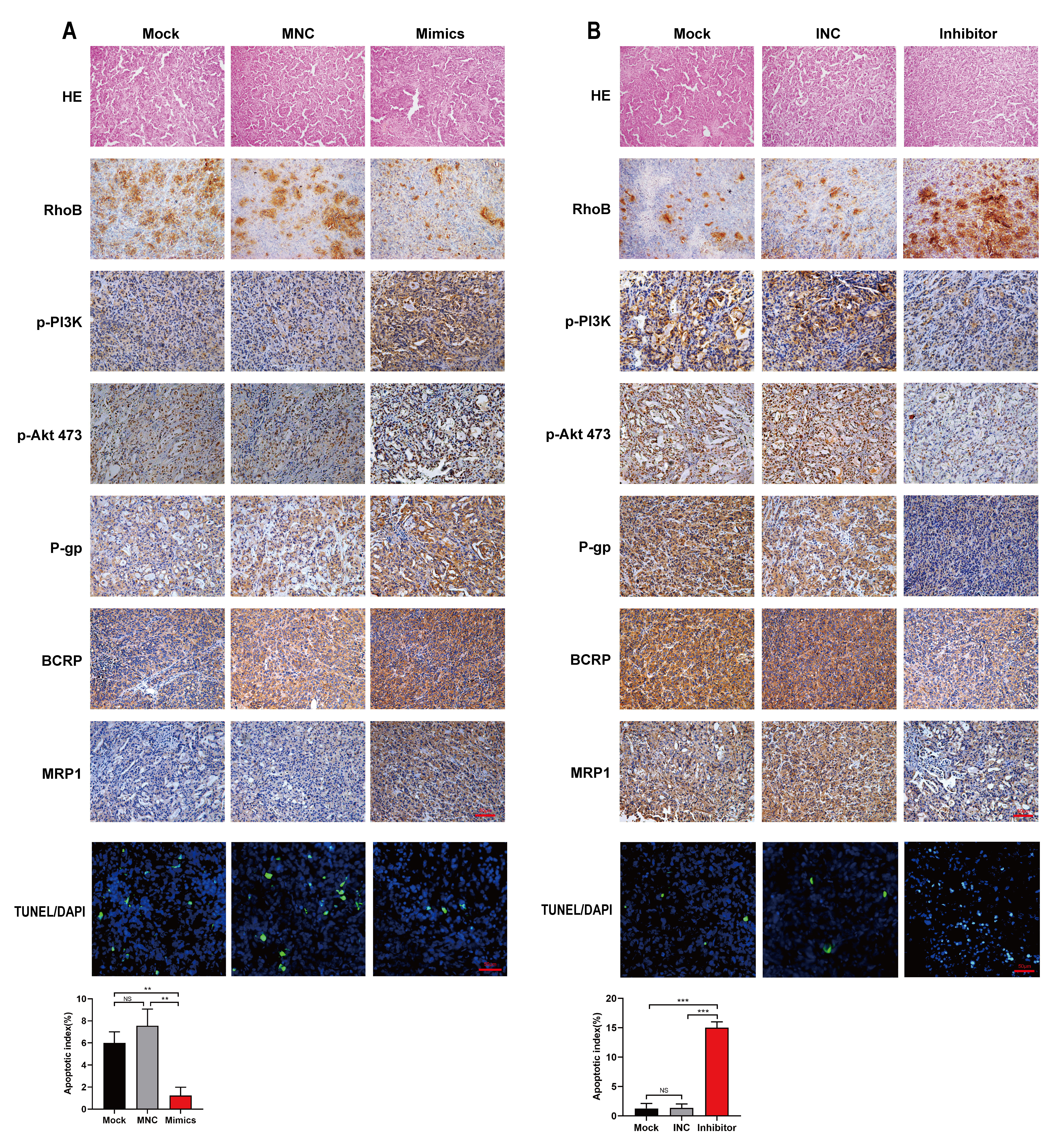

Supplement: Supplementary file 2 — Additional file 2: Supplementary Figure 1. Hsa-miR-3178 restrainsexpression of RhoB and promotes expression of phosphorylated-PI3K andphosphorylated-Akt 473 in vivo. (A, B) IHC on theexpression of RhoB, phosphorylated-PI3K, phosphorylated-Akt 473, P-gp, BCRP andMRP1 in the indicated groups. And the TUNEL+ cell proportion in the indicatedgroups. Scale bar, 50 μm.Data are expressed as mean ±SD from three independentexperiments. **P < 0.01; ***P < 0.001. IHC: immunohistochemistry. TUNEL:Terminal-deoxynucleoitidyl Transferase Mediated Nick End Labeling. [file 12943_2022_1587_MOESM2_ESM.jpg]
